# Pneumomediastinum Associated with Pneumopericardium and Epidural Pneumatosis

**DOI:** 10.1155/2014/275490

**Published:** 2014-05-13

**Authors:** Ozlem Bilir, Ozcan Yavasi, Gokhan Ersunan, Kamil Kayayurt, Baris Giakoup

**Affiliations:** Department of Emergency Medicine, Recep Tayyip Erdogan University, Rize Research and Training Hospital, 53020 Rize, Turkey

## Abstract

Spontaneous pneumomediastinum is a relatively rare benign condition. It may rarely be associated with one or combination of pneumothorax, epidural pneumatosis, pneumopericardium, or subcutaneous emphysema. We present a unique case with four of the radiological findings in a 9-year-old male child who presented to our emergency department with his parents with complaints of unproductive cough, dyspnea, and swelling on chest wall. Bilateral subcutaneous emphysema was palpated on anterior chest wall from sternum to midaxillary regions. His anteroposterior and lateral chest radiogram revealed subcutaneous emphysema and pneumomediastinum. His thorax computed tomography to rule out life-threatening conditions revealed bilateral subcutaneous, mediastinal, pericardial, and epidural emphysema without pneumothorax. He was transferred to pediatric intensive care unit for close monitorization and conservative treatment. He was followed-up by chest radiographs. He was relieved from symptoms and signs around the fifth day and he was discharged at the seventh day. Diagnosis of pneumomediastinum is often made based on physical findings and plain radiographs. It may not be as catastrophic as it is seen. Close cardiopulmonary monitorization is mandatory for complications and accompanying conditions. Most patients with uncomplicated spontaneous pneumomediastinum respond well to oxygen and conservative management without any specific treatment.

## 1. Introduction

Pneumomediastinum, pneumopericardium, epidural pneumatosis, and subcutaneous emphysema are disorders characterized by the presence of free air or gas in the related spaces. They are usually self-limited conditions unless tension pneumomediastinum, tension pneumothorax, air tamponade and cardiac herniation, and esophageal rupture accompany these benign disorders [1, 2]. The combination of pneumomediastinum with epidural pneumatosis, pneumopericardium, and subcutaneous emphysema is a very rare condition. We present a unique case with four of the radiological findings in a 9-year-old male child.

## 2. Case Report

**Figure 1:**
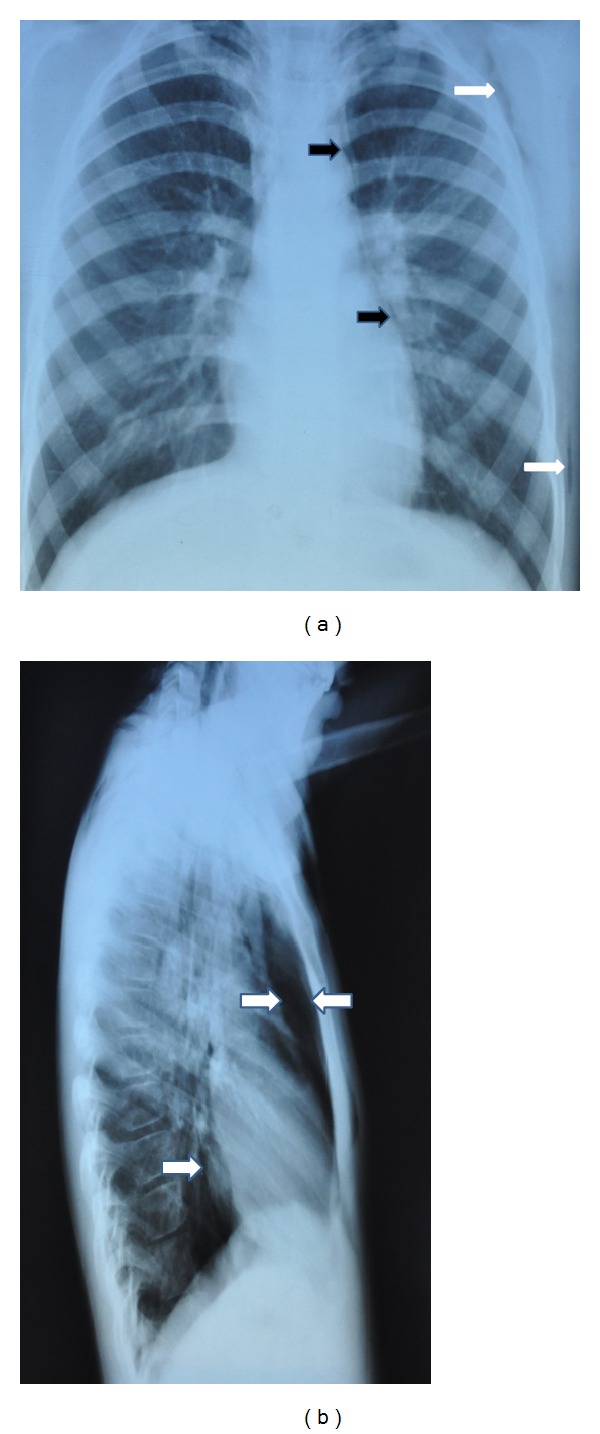
(a) Anteroposterior chest radiograph showing thin radiolucent line outlining aortic root and left heart border (black arrows) and subcutaneous emphysema (white arrows). (b) Lateral chest radiograph showing retrosternal emphysema (between arrows) and radiolucent line outlining posterior border of the heart.

**Figure 2:**
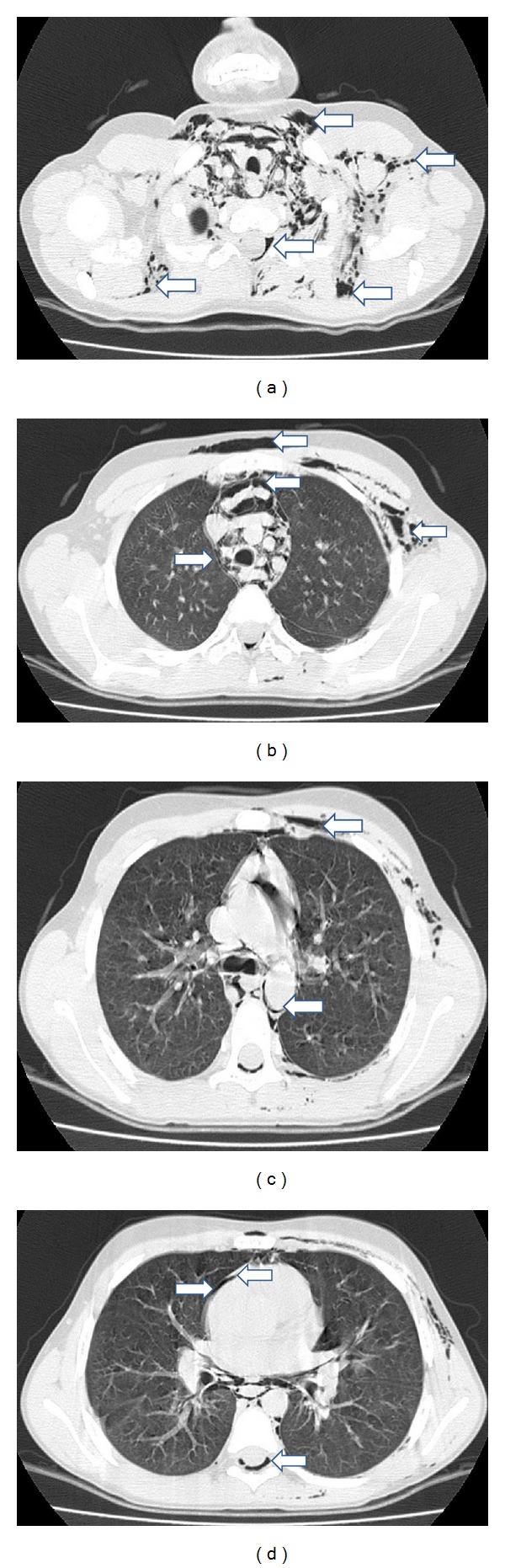
Chest computed tomography of the patient. (a) Free air in the soft tissues of the neck, subcutaneous emphysema, and air in epidural space of cervical vertebra. (b) Subcutaneous emphysema in the soft tissues of the left hemithorax and anterior chest wall as well as mediastinal emphysema surrounding mediastinal structures. (c) Free air surrounding mediastinal vascular structure and subcutaneous emphysema. (d) Pneumopericardium and air in the epidural space of the thoracic vertebra.

A 9-year-old male child with complaints of unproductive cough, dyspnea, and swelling on chest wall presented to our emergency department with his parents. He did not state an obvious chest pain. His complaints had started 12 hours ago. He does not have any trauma history. His history and family history were unremarkable and he had never been diagnosed with bronchial asthma before. He was conscious and in sitting position because of dyspnea and had expiratory wheezing. His weight was 33 kg (between 75th and 90th percentiles) and height was 138 cm (between 75th and 90th percentiles). His vital signs were blood pressure, 100/60 mmHg; pulse rate, 140 beats/minute; respiratory rate, 45/minute; temperature, 36,9°C; and oxygen saturation, 80% at room air. Bilateral subcutaneous emphysema was palpated on anterior chest wall from sternum to midaxillary regions. On auscultation he had rhonchus on both hemithoraces, prolonged expirium, and Hamman's sign. An intravenous line was inserted and 8 L/minutes oxygen and nebulized salbutamol were started. Arterial blood gas analysis revealed pH, 7.43; PaCO_2_, 45 mmHg; and PaO_2_, 75 mmHg. Other laboratory findings were as follows: white blood cell count, 5.40 K/uL; neutrophil count, 1.52 K/uL; hemoglobin, 13.5 mg/dL; platelet, 266 K/uL; C-reactive protein, 0.55 mg/dL; and immunoglobulin E, 419 IU/mL (normal range; 0–165 IU/mL). His saturation was elevated to 90%. His electrocardiogram showed sinus tachycardia. His anteroposterior and lateral chest radiogram revealed subcutaneous emphysema and pneumomediastinum (Figure 1). His thorax computed tomography revealed bilateral subcutaneous, mediastinal, pericardial, and epidural emphysema without pneumothorax (Figure 2). He consulted a pediatric surgeon and was transferred to pediatric intensive care unit for close monitorization and conservative treatment. He was administered oxygen, cold-vapor, nebulized salbutamol, and prophylactic antibiotics (ampicillin-sulbactam). He was followed-up by chest radiographs. He was relieved from symptoms and signs around the fifth day and he was discharged at the seventh day. His family stated that he usually had cough and dyspnea without fever in spring and summer but they did not seek any treatment before. He was referred to pediatric allergy department and diagnosed with seasonal allergic asthma one month later. He did not have any asthma attack during a 5-month period. His family migrated to another city and the patient lost follow-up.

## 3. Discussion

Pneumomediastinum, also known as mediastinal emphysema, is defined as the presence of air or other gases in the mediastinum [3, 4]. It is a rare and generally benign self-limited condition first reported in the literature in 1939 by Hamman [1]. Pneumomediastinum can be categorized as spontaneous or traumatic. There is a bimodal peak incidence in children aged less than 4 years and in those aged 15–18 years. Infection is the leading cause of spontaneous pneumomediastinum in preschool and middle childhood but in adolescents it is more commonly idiopathic [5]. In the pediatric age group, the most common triggers of pneumomediastinum are asthma exacerbation, respiratory infections, vomiting, situations reproducing the Valsalva maneuver (e.g., shouting, coughing), and intense physical exertion [1, 6]. The rate of spontaneous pneumomediastinum among children presenting for emergency treatment of asthma is between 0.3 and 5% [7, 8].

Pneumopericardium can be an occasional complication of pneumothorax and pneumomediastinum and is defined as a collection of air or gas in the pericardial cavity and was first described by Bricheteau in 1844 [9]. Many cases have since been reported, mostly due to blunt or penetrating chest injuries in adults and due to respiratory distress syndrome and positive pressure mechanical ventilation in infants [9–11]. In 37% of patients with pneumopericardium, cardiac tamponade may develop and pneumopericardium increases mortality to 58% [9].

Epidural pneumatosis following lumbar puncture and epidural analgesia is well known. It has also been reported following trauma, excessive exercise, and chest tube placement. Epidural pneumatosis secondary to pneumomediastinum is a benign entity that does not cause neurological deficit and should not be a cause of excessive concern [12].

Pneumomediastinum is usually associated with activities that raise the intrathoracic pressure. In these cases it is thought that excessive intra-alveolar pressures lead to rupture of perivascular alveoli. The free air then dissects from the ruptured alveoli along the bronchovascular sheaths toward the mediastinum to produce spontaneous pneumomediastinum. This sequence of events is known as the “Macklin effect” [13]. Accumulated mediastinal gas may decompress along cervical fascial planes into the subcutaneous tissue to produce subcutaneous emphysema. Epidural pneumatosis occurs when free air tracks through fascial planes in the brachial plexus, axillary arteries, intercostal nerves, or intervertebral foramina [14].

The pathognomonic feature of subcutaneous emphysema is crepitus on palpation. The clinical presentation of spontaneous pneumomediastinum, pneumopericardium, and subcutaneous emphysema is chest pain, dyspnea, Hamman's sign, and crepitus on palpation [7, 15]. Hamman's sign is a crunching, rasping sound caused by the heart beating against air-filled tissues, heard during cardiac systole often with a decrease in heart sounds, and is pathognomonic for pneumomediastinum. Our patient was brought to the emergency department with chest pain and swelling on anterior chest wall following coughing spells. He had crepitus and Hamman's sign was heard.

Although computed tomography is more sensitive than plain chest radiographs in detecting spontaneous pneumomediastinum, in most of the times a clear anamnesis and anteroposterior and lateral chest radiographs including cervical region confirm diagnosis. Tomography should be reserved for evaluation of suspected underlying lung diseases and accompanying conditions [16].

An association between a pneumomediastinum, a pneumopericardium, a pneumothorax, and a skin emphysema is extremely rare in children and young adults [17]. Spontaneous pneumomediastinum is often associated with subcutaneous air, and pneumothorax is present in approximately 50% of the cases [18]. In our case, pneumomediastinum was associated with pneumopericardium, epidural pneumatosis, and subcutaneous emphysema.

Although pneumomediastinum is a benign self-limited condition, the risk of tension pneumomediastinum or pneumothorax justifies close clinical follow-up. Accompanying esophageal rupture carries a 70% risk of mortality, despite operative intervention [1]. The main potential life-threatening complications of pneumopericardium are air tamponade and cardiac herniation. Air tamponade is similar in pathophysiology to classical cardiac tamponade. Cardiac herniation can also lead to significant cardiac pump dysfunction [2].

Spontaneous pneumomediastinum in children should be managed under intensive cardiopulmonary monitoring. The treatment of spontaneous pneumomediastinum depends upon whether or not there are complications. Close cardiopulmonary monitorization is mandatory for complications and accompanying conditions. Uncomplicated spontaneous pneumomediastinum is managed conservatively with analgesia, rest, and avoidance of maneuvers that increase pulmonary pressure (Valsalva or forced expiration, including spirometry) [19]. Our patient was also treated conservatively with oxygen, cold-vapor, nebulized salbutamol, and prophylactic antibiotics. He was not given analgesic as there was not an obvious complaint of chest pain.

We presented this case because it was an underdiagnosed case of asthma. Our patient manifested pneumomediastinum in the first asthma attack. When it is triggered by asthma as in our case, effective asthma therapy reduces alveolar overinflation, stopping the flow of air into the mediastinum and permitting the absorption of mediastinal and subcutaneous air within 5–7 days. We suggest to further evaluate the children for asthma and its complications such as pneumomediastinum who present with cough and dyspnea to the emergency departments. Diagnosis of pneumomediastinum in young asthmatic children is not always easy as they are not often able to complain about chest pain, which is a significant clinical sign in adults.
